# Gasless robot-assisted transaxillary hemithyroidectomy (RATH): learning curve and complications

**DOI:** 10.1186/s12893-024-02366-7

**Published:** 2024-03-02

**Authors:** Pengfei Xu, Qi Fang, Junhao Mai, Zheng Zhao, Fei Cao, Di Wu, Xuekui Liu

**Affiliations:** 1 Department of Head and Neck Surgery, State Key Laboratory of Oncology in South China, Collaborative Innovation Center for Cancer Medicine, Sun Yat-sen University Cancer Center, 651 Dongfeng East Road, Guangzhou, Guangdong 510060 P. R. China; 2 Department of Breast and Thyroid Surgery, Guangzhou Panyu Central Hospital, 8 Fuyu East Road, Guangzhou, Guangdong 511400 P.R. China

**Keywords:** Robot-assisted, Thyroid, Transaxillary, Learning curve, Complication

## Abstract

**Purpose:**

Gasless robot-assisted transaxillary hemithyroidectomy (RATH) is regarded as an alternative surgical option for thyroid operations. However, the associated steep learning curve is a clinical concern. This study evaluated the learning curve of RATH for surgeons without experience of endoscopic surgery and the early surgical outcomes of RATH.

**Methods:**

We conducted a retrospective study of patients who underwent gasless RATH and conventional hemithyroidectomy (CH) at Sun Yat-sen University Cancer Center, Guangzhou, China, from June 2021 to August 2022. The learning curve and early surgical outcomes of gasless RATH were evaluated. And the early surgical outcomes of gasless RATH were compared to CH.

**Results:**

In total, 105 patients who underwent gasless RATH and 104 patients who underwent CH were matched and assessed. The cumulative sum techniques (CUSUM) analysis showed that the peak point of gasless RATH operative time occurred at the 31st case. No clear single peak was identified in the CUSUM plot for drainage amount and blood loss. No significant difference in perioperative complications was observed between these two groups. Moreover, the number of postoperative patients who got sense of thyroid area traction were fewer in the gasless RATH group (*n* = 11, 10.5%) than in the CH group (*n* = 32, 30.8%).

**Conclusion:**

Gasless RATH can be considered as an alternative approach to the conventional open procedure, as it is an easy remote access technique, with shorter learning curves and certain advantage such as less sense of thyroid area traction.

## Introduction

Since its introduction by Kocher in the late 1880s, the transcervical incision has been the favored surgical approach to the thyroid and parathyroid glands. This incision design is obviously advantageous, as it provides the surgeon with excellent exposure and direct access to the central neck [1]. Some advances such as minimally invasive video-assisted thyroidectomy (MIVAT) [2], have gained popularity as a method to reduce incision size. However, regardless of how well the incision is closed, a scar of different prominence is inevitable. The impact of such a scar varies among patients, and it may have diverse implications on the quality of life, esthetic enjoyment, and social interactions.

Remote access surgical methods originated in the past century and are currently employed in a wide range of surgical specialties, including abdominal, thoracic, and, more recently, head and neck surgery [3]. Endoscopic and robotic procedures are already well-established treatment options in these fields. Both these approaches use well-hidden incisions and effectively improve the patients’ quality of life as well as being esthetically acceptable. Moreover, da Vinci robot’s expanded field of view, 3D view, tremor filtering system, endo wrist technology, and multiarticulations of the arms (7 degrees of freedom) provide greater range of motion and precision than the endoscopic approach, while also minimizing collisions between the robotic arms and camera [4].

At present, the two commonly used robotic approaches are the gasless transaxillary approach and the bilateral axillo-breast approach (BABA) [5], both of which have advantages and disadvantages. The robotic BABA procedure enables surgeons to perform complete thyroid resection with good visualization. Robotic BABA provides a view similar to conventional surgery. This approach has the advantage of being able to perform a total thyroidectomy with good surgical completeness [6]. However, due to insufficient entry angle and the presence of the manubrium and clavicle, the BABA approach may encounter challenges in clearing lymph nodes in the central region below the manubrium [7]. Further, CO_2_-related complications such as subcutaneous emphysema, hypercapnia, respiratory acidosis, and cerebral edema are also limitations of the BABA approach. The robot-assisted transaxillary thyroidectomy (RATT) eliminates the need for insufflation, hence reducing the risks of such CO_2_-related complications that may occur with traditional endoscopic techniques [3]. However, an adequate angle of visualization with RATT allows for a more thorough central neck dissection. Therefore, we chose the transaxillary approach in our study.

The aim of this study was to assess the time required for training in gasless robot-assisted transaxillary hemithyroidectomy (RATH) and compare the complications of gasless RATH and open thyroid surgery.

## Materials and methods

### Study design

A retrospective cohort study was designed including all consecutive cases that underwent conventional thyroid hemithyroidectomy and gasless RATH from June 2021 to August 2022 at the Department of Head and Neck Surgery, Sun Yat-sen University Cancer Center, Guangzhou, China. Depending on the surgical approach, patients were divided into two groups: (1) gasless RATH and (2) transcervical conventional approach.

The inclusion criteria for both groups were: (1) those who underwent ipsilateral central compartment node dissection; (2) those who were operated for the first time; and (3) cases wherein all procedures were performed by a principal surgeon with no experience of endoscopic surgery or robot-assisted surgery. (4) those without lymph node metastasis or with only VI zone lymph node metastasis and a diameter less than 3 cm. We considered the following surgical complications: recurrent laryngeal nerve injuries (temporary or permanent, 3 months as the cut-off time), hypocalcemia (symptomatic or requiring calcium supplementation), seroma, hematoma, surgical site infection, thyroid area traction (assessed one month postoperatively through a yes or no questionnaire), local nerve hypoesthesia, and brachial plexus palsy (gasless RATH only). Before performing the RATH for the first time, the chief surgeon has been trained in robotic surgery and obtained a qualification certificate. At the same time, the first 5 operations were all completed under the guidance of surgeons with rich experience in RATH. Ethical approval for the study was obtained from the institutional ethical committee of Sun Yat-sen University Cancer Center (XJS2022-007-01). All patients gave informed consent to the above.

### Operative method

**Fig. 1:**
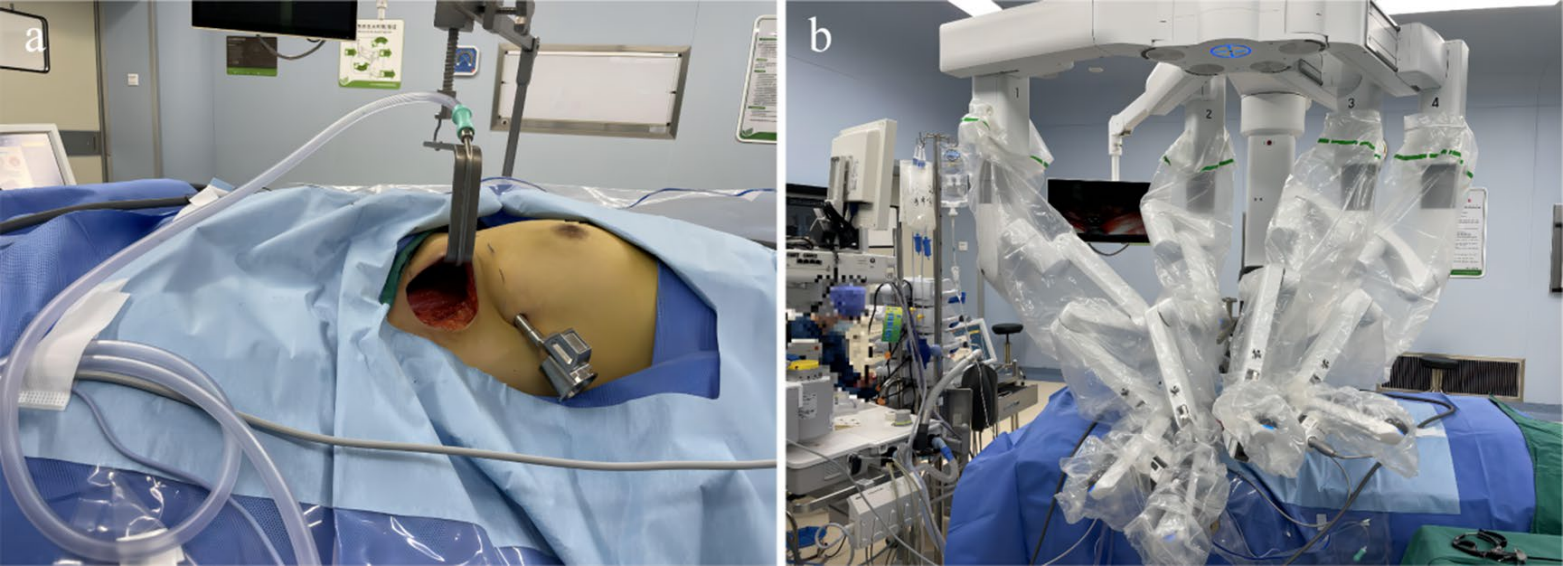
(**a**) Establishment of workspace. (**b**) Position of arms of the da Vinci robot

Gasless RATH: The patient was placed supine with the neck slightly extended. The arm on the side of the access was abducted to 100°, supinated, and the elbow flexion was 30°. The arm was placed on an arm table. The incision, measuring 6–8 cm, started from the intersection of the front axillary line and the axillary crease, and turned vertically downward as it travelled along the axillary crease to the midaxillary line. A < 1 cm auxiliary incision was made in the midaxillary line 3–5 cm under the main incision. An endoscope was used to build the surgical passage; this passage can also be established under direct vision. A subcutaneous flap was raised over the pectoralis major muscle all the way to the neck to provide the working area. The working space was maintained by specially developed retractors (Fig. 1a). All arms of the da Vinci robot were introduced, and grasping forceps, a three-dimensional endoscope, a Maryland dissector, and a harmonic scalpel were placed in the corner of the main incision left to right respectively (the robotic arm closest to the auxiliary incision passed through the poke hole, Fig. 1b). The upper pedicle was cut first, after which the thyroid lobe was retracted medially. The surgeon began by cutting or, alternately, bluntly using the Maryland dissector to dissect the tracheoesophageal groove to identify the recurrent laryngeal nerve. We located and prepped the parathyroid glands. The thyroid lobe was sliced using a harmonic scalpel once the crucial features had been located, ipsilateral central neck dissection of lymph nodes was simultaneously performed, and the specimen was finally extracted.

**Fig. 2:**
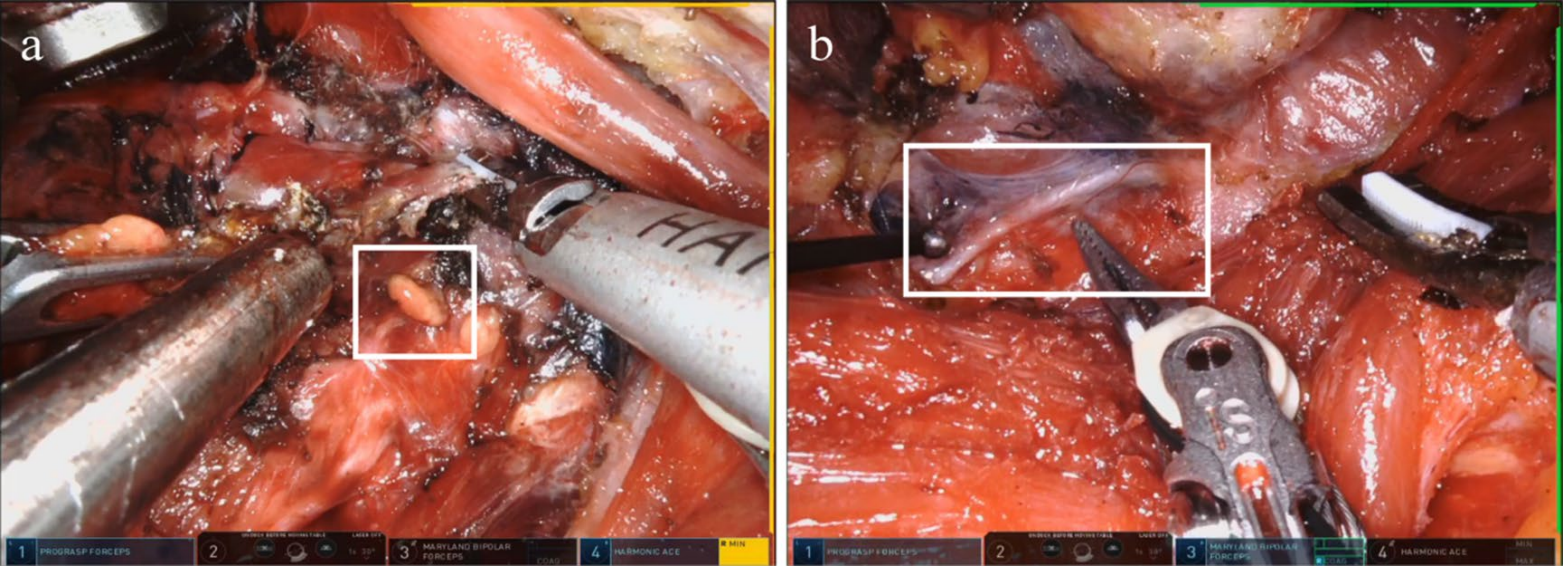
The endoscopic view of the operation procedure (left). (**a**) Superior parathyroid (**b**) Recurrent nerve

Conventional hemithyroidectomy (CH): The patient was placed supine with their neck extended during open surgery. The subplatysmal flap was dissected via a 4–5-cm-long cervical incision. The thyroid gland was visible, as the midline between the strap muscles was split. From the superior pole to the inferior pole, the thyroid gland was dissected. To protect the parathyroid glands (Fig. 2a), the inferior thyroid arteries were individually tied up near the thyroid gland. The ipsilateral laryngeal nerve’s full cervical path was traced and recorded (Fig. 2b).

### Cumulative sum (CUSUM) analysis

CUSUM is a change-detection analytical approach that has been used to quantitatively evaluate the learning curve. The cumulative differences between each person and the mean value across all instances were calculated using CUSUM. All patients in this research were arranged chronologically. The average operating time was *u*, whereas the operating time for the $i$_*th*_ patient was ${S}_{i}$. The difference between the operative time for the first patient (*S*_*1*_) and mean operative time ($u$) was the CUSUM for the first patient. The CUSUM for the second patient was the CUSUM of the first patient plus the difference between the operative time for the second patient (*S*_*2*_) and the mean operative time ($u$). This procedure was repeated up to the last instance, at which point the equation shown below would result in the CUSUM being equal to zero.

$${\bf{CUSUM}}\,{\rm{ = }}\,\sum\nolimits_{i = 1}^n {\left( {{S_i}\, - \,u} \right)}$$

The CUSUM chart was used to visually depict the findings. The trend of data departure from the mean value was indicated by the CUSUM value. In the CUSUM chart, an upward slope meant moving away from the goal value and a downward slope implied moving closer to the target value. The point at which the slope changed direction showed skill competence. Similarly, the CUSUM for the quantity of postoperative drainage was determined. The moving average may get rid of small deviations and emphasize long-term patterns in the data, giving the graph a “smoothing” curve. To examine the operation’s duration, blood loss, and drainage volume accurately, we employed a moving average of 5.

$${\rm{Moving}}\,{\rm{average}}\,i\, = \,{{{S_i}\, + \,{S_{i + 1}}\, + \,{S_{i + 2}}\, + \,{S_{i + 3}}\, + \,{S_{i + 4}}} \over 5}$$

Operative time was defined as the total work time of the robotic arm. The postoperative drainage amount was defined as the summation of drainage volume in the first two postoperative days. Patients discharged with a drainage tube were required to record their daily drainage.

### Statistical analysis

Following the Shapiro–Wilk test of normality, a statistical comparison between the two surgical groups was made, as well as correlations between variables, using the chi-square test and two-tailed Fisher’s exact test for categorical data and the Mann–Whitney U test and Student’s *t*-test for continuous data. The statistical software used was the SPSS version 21 (IBM Corporation, Armonk, NY, USA). A value of *p* < 0.05 was considered to indicate statistically significant differences.

## Results

### Patients

A total of 105 individuals who underwent gasless RATH were included in the research. Their mean age was 36.2 (range: 19–54) years, and 77 (73.3%) were women. The subjects in this group had a mean body mass index (BMI) of 22.7 ± 3.5 (range: 16.9–33.6) kg/m^2^. There were no conversions to open surgery. The mean tumor size was 1.0 ± 0.6 (range: 0.2–3.5) cm, and 8 (7.6%) cases were benign. The mean postoperative length of stay was 2.1 ± 0.8 (range: 1–5) days.

In the CH group, 104 individuals were included. Their mean age was 43.9 (range: 25–71) years, and 77 (74.0%) were women. The subjects in this group had a mean BMI of 23.9 ± 3.4 (range: 15.4–33.3) kg/m^2^. The mean tumor size was 0.9 ± 6.7 (range: 0.3–5.5) cm, and 3 (2.9%) cases were benign (Table 1). Only one patient had a tumor size > 4 cm. This patient underwent unilateral lobectomy, because frozen section examination of the operative specimen was unable to determine whether the lesion was benign or malignant. However, postoperative pathology confirmed that the tumor was malignant. The patient did not undergo supplementary surgery. TNM staging did not differ between groups. The mean postoperative length of stay was 1.0 ± 0.4 (range: 1–4) days. All patients in this study received unilateral lobectomy and underwent unilateral center neck dissection.

**Table 1:** Baseline characteristics of the study populations

Characteristic	RATH (range, %) (*N* = 105)	CH (range, %) (*N* = 104)	P-value
Age (year)	36.2 ± 8.1 (19–54)	43.9 ± 11.8 (25–71)	**0.000**
Sex (F:M)	2.75 (73.3):1 (26.7)	2.85 (74.0):1 (26.0)	0.908
Mean BMI (kg/m^2^)	22.7 ± 3.5 (16.9–33.6)	23.9 ± 3.4 (15.4–33.3)	**0.009**
Tumor size (cm)	1.0 ± 0.6 (0.2–3.5)	0.9 ± 6.7 (0.3–5.5)	0.949
Final Pathology (%)			
Benign	8 (7.6)	3 (2.9)	0.125
Malignant	97 (92.4)	101 (97.1)	
Pathological T classification (%)			
T1a	67 (69.1)	68 (67.3)	
T1b	21 (21.6)	27 (26.7)	
T2	7 (7.2)	5 (5.0)	
T3	0 (0)	1 (1.0)	
T4a	2 (2.1)	0 (0)	0.404
Pathological N classification (%)			
N0	67 (69.1)	68 (67.3)	
N1a	30 (30.9)	33 (32.7)	0.792
Postoperative length of stay (day)	2.1 ± 0.8 (1–5)	1.0 ± 0.4 (1–4)	**0.000**

*Abbreviations* RATH, gasless robot-assisted transaxillary hemithyroidectomy; CH: conventional hemithyroidectomy; BMI, body mass index

### Learning curve

**Fig. 3:**
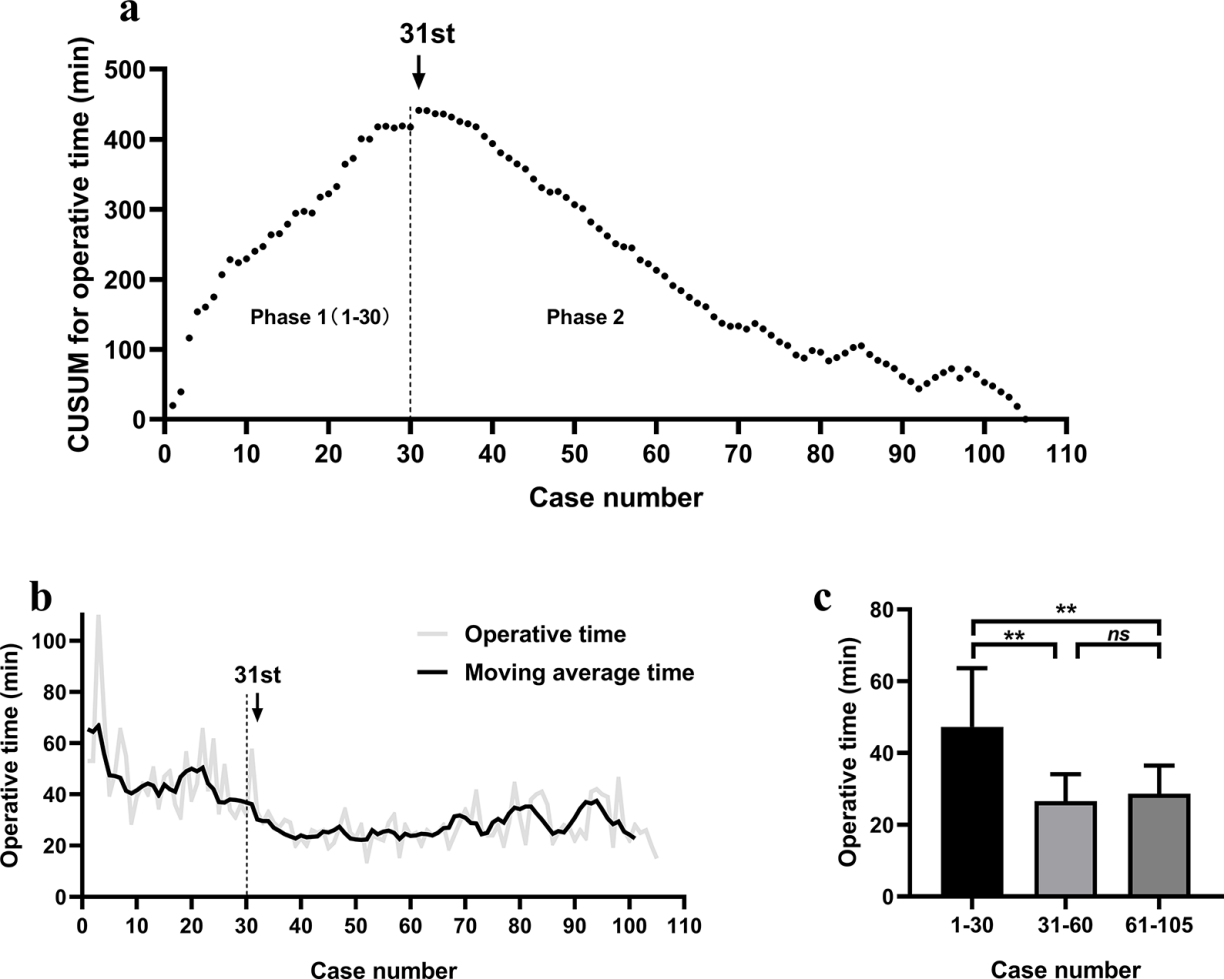
(**a**) Cumulative sum (CUSUM) control chart for the robot-assisted transaxillary hemithyroidectomy operative time. (**b**) Moving average for the operative time. (**c**) Mean operative time by group. *Abbreviations*: *, *P* ≤ 0.05; **, *P* ≤ 0.01, *ns*, *P*>0.05

In our study, a single, high-volume (> 1000) surgeon who typically performs conventional hemithyroidectomy without prior experience of robot-assisted operation conducted all surgeries. We utilized the CUSUM plot to examine learning curves of for the gasless RATH methods. The results showed that the CUSUM value of operative time reached its maximum point that the slope change from ascending to descending in the 31st case (Fig. 3a). Based on the results of the learning curve analysis, all patients were divided into three phases: phase 1 (1–30 cases), phase 2 (31–60 cases), and phase 3 (61–105 cases). The moving average curve approach was used; the line chart shows a similar trend as the CUSUM plot (Fig. 3b). A comparison of the average operative times of phases 1, 2, and 3 was also done. A substantial decrease in average operative time was detected between phase 1 (47.3 ± 16.3 min) and phase 2 (26.5 ± 7.6 min; *P* = 0.000) but such decrease was not observed between phase 2 and 3 (28.6 ± 7.8 min; *P* = 0.255, Fig. 3c). The slope remained smooth, indicating relatively stable average operative time over the phase 2 and 3 timeframes (Fig. 3b).

**Fig. 4:**
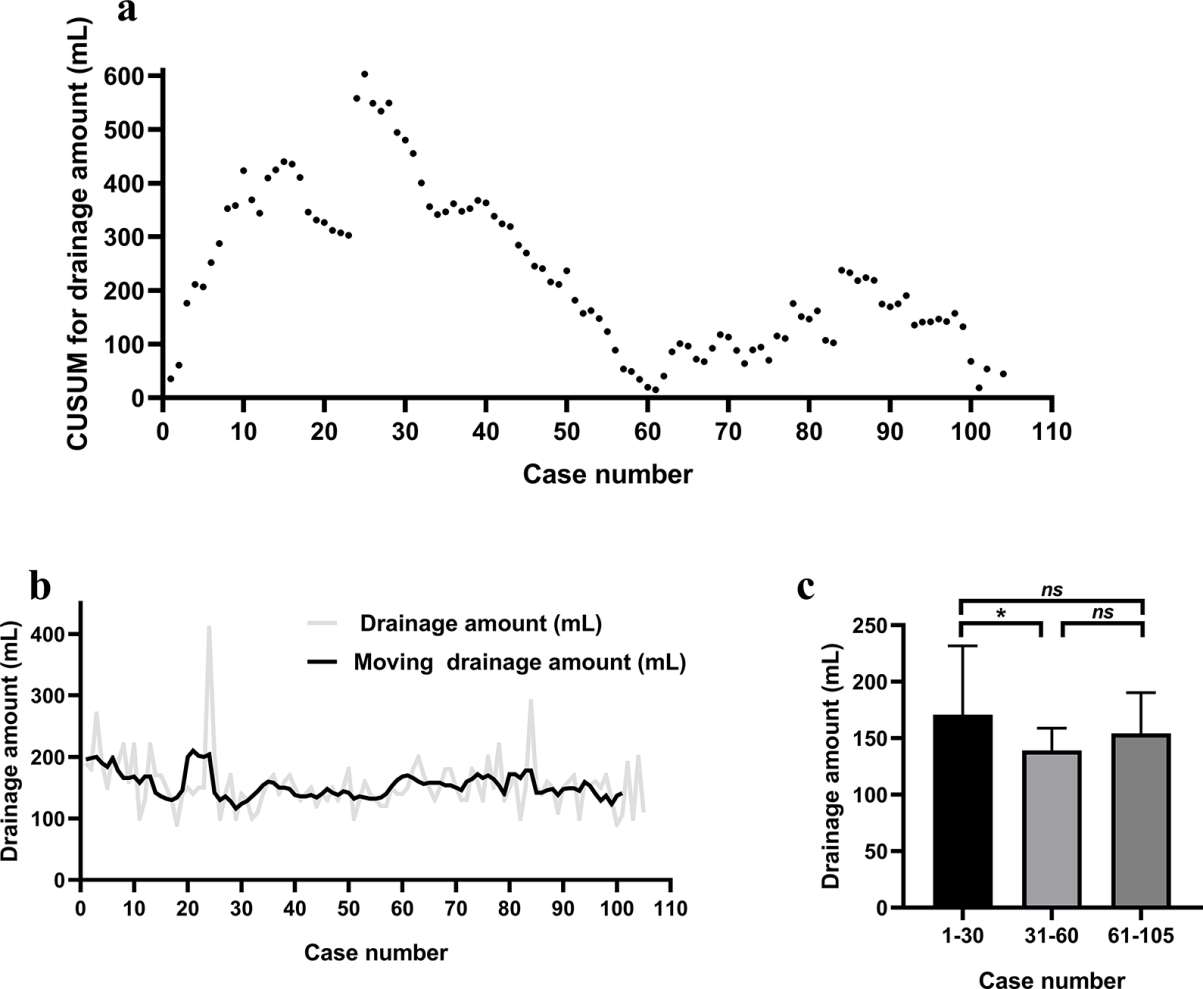
(**a**) Cumulative sum (CUSUM) control chart for the robot-assisted transaxillary hemithyroidectomy drainage amount. (**b**) Moving average for the drainage volume. (**c**) Mean drainage amount by group. *Abbreviations*: *, *P* ≤ 0.05; **, *P* ≤ 0.01, *ns*, *P*>0.05

The CUSUM plot for drainage volume did not show a clear single peak (Fig. 4a). The moving average curve showed that a decrease in average drainage volume was detected between phase 1 and phase 2 (Fig. 4b). Comparisons of the drainage volume of phases 1, 2, and 3 were carried out. The drainage volume of phase 1 (170.7 ± 61.1 mL) was significantly more than that of the phase 2 (139.3 ± 19.5 mL, *P* = 0.004, Fig. 4c). The difference in the drainage volume between phase 2 and phase 3 (154.2 ± 36.1 mL) was not statistically significant (*P* = 0.097, Fig. 4c).

**Fig. 5:**
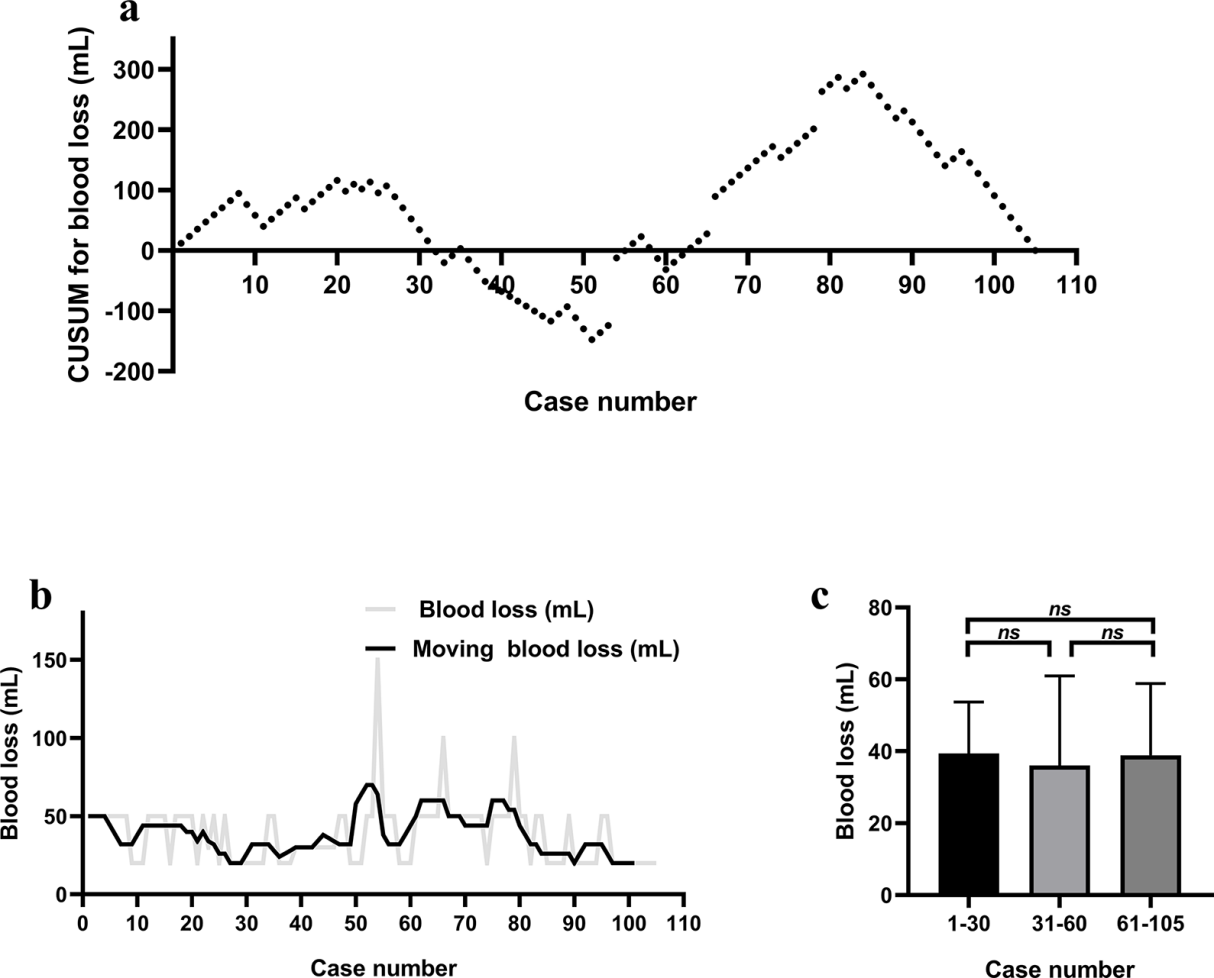
(**a**) Cumulative sum (CUSUM) control chart for the robot-assisted transaxillary hemithyroidectomy blood loss. (**b**) Moving average for the blood loss. (**c**) Mean blood loss by group. *Abbreviations*: *, *P* ≤ 0.05; **, *P* ≤ 0.01, *ns*, *P*>0.05 **Consent to Publication**: not applicable

The CUSUM plot for blood loss did not show a clear single peak (Fig. 5a). The moving average curve showed a smooth continuous slope (Fig. 5b). The difference in blood loss between phase 1 (39.3 ± 14.4 mL), phase 2 (36.0 ± 25.0 mL), and phase 3 (38.9 ± 20.0 mL) was not statistically significant (*P* = 0.778, Fig. 5c).

### Surgical outcomes and complications

Comparisons of both study groups focused on cases characteristics, operative outcomes, and complications are shown in Table 2. Operative time was, as expected, longer in the gasless RATH group (125.4 ± 28.7 vs. 49.4 ± 16.5 min, *P* < 0.001). The drainage volume for 48 h in the gasless RATH group was more than that in the CH group (154.7 ± 42.9 vs. 95.3 ± 30.1 mL, *P* < 0.001). The retrieved lymph node of the gasless RATH group was significantly less than that of the CH group (3.3 ± 2.7 vs. 4.4 ± 3.4, *P* = 0.009), but the difference in lymph node metastasis between the two groups was not significant (30.5% vs. 31.7%, *P* = 0.845).

**Table 2:** Comparison of surgical outcomes between the two phases

Characteristic	RATH (range, %) (*N* = 105)	CH (range, %) (*N* = 104)	P-value
Operative time (min)	125.4 ± 28.7 (79–215)	49.4 ± 16.5 (23–110)	**0.000**
Drainage amount for 48 h (mL)	154.7 ± 42.9 (90–410)	95.3 ± 30.1 (15–160)	**0.000**
Retrieved lymph node	3.3 ± 2.7 (0–12)	4.4 ± 3.4 (0–19)	**0.009**
Lymph node metastatic	32 (30.5)	33 (31.7)	0.845
Postoperative calcium (mmol/L)	2.09 ± 0.08 (1.87–2.27)	2.11 ± 0.09 (1.85–2.39)	**0.018**
Low postoperative calcium (< 2.0 mmol/L)	13 (12.4)	8 (7.7)	0.260
Postoperative PTH (pg/mL)	31.1 ± 9.5 (10.3–68.6)	27.4 ± 10.0 (3.3–53.0)	**0.006**
Low Postoperative PTH (< 15 pg/mL)	3 (2.9)	7 (6.7)	0.190
Permanent hypocalcemia	0 (0.0)	0 (0.0)	-
Vocal cord palsy			
Transient	11 (10.5)	8 (7.7)	0.484
Permanent	1 (0.95)	0 (0.0)	1.000
Hematoma (reoperation)	1 (0.95)	0 (0.0)	1.000
Trachea injury	1 (0.95)	0 (0.0)	1.000
Wound seroma	0 (0.0)	1 (0.96)	0.498
Chyle fistula (observation)	0 (0.0)	0 (0.0)	-
Wound infection	1 (0.95)	0 (0.0)	1.000
Brachial plexus neuropraxia	0 (0.0)	-	-

*Abbreviations* RATH, gasless robot-assisted transaxillary hemithyroidectomy; CH: conventional hemithyroidectomy; PTH, parathyroid hormone

There were 13 (12.4%) and 8 (7.7%) patients, respectively, with transient low postoperative calcium (< 2.0 mmol/L) levels in RATH and CH groups, but this difference was not statistically significant. There were 3 (2.9%) and 7 (6.7%) patients, respectively, with transient low postoperative parathyroid hormone (PTH) (< 15 pg/mL) levels in RATH and CH groups, but this difference was not statistically significant. The mean postoperative calcium in the gasless RATH group (2.09 ± 0.08 mmol/L) was significantly lower than that in the CH group (2.11 ± 0.09 mmol/L, *P* = 0.018), but the mean postoperative PTH of the gasless RATH group (31.1 ± 9.5 pg/mL) was significantly higher than that in the CH group (27.4 ± 10.0 pg/mL, *P* = 0.006). Patients did not receive calcium or vitamin D supplements. No patients appeared to have permanent hypocalcemia.

Postoperative complications early after the operation did not differ significantly between the two groups (Table 2). In gasless RATH group, we observed 11 (10.5%) temporary vocal cord paresis, 11 (10.5%) postoperative thyroid area traction, 1 (0.95%) permanent vocal cord paresis, 1 (0.95%) hematoma (reoperation), 1 (0.95%) trachea injury, and 1 (0.95%) infection. All temporary vocal cord paresis were resolved within 3 months. One patient underwent resection of the left recurrent laryngeal nerve for direct tumor invasion. We recommended a total thyroidectomy and iodine therapy after the operation, but the patient rejected our suggestion and had a strong will to preserve the contralateral thyroid lobe. No other patients had clinical evidence of permanent recurrent laryngeal nerve injury. The drainage volume of one patient exceeded 200 mL within 1 h after the operation, and emergency incision hemostasis was performed. The bleeding site was the external jugular vein, not the thyroid area. During the operation of one patient, the thyroiditis was found to form a mass adherent to the trachea. We removed the lobe completely by scraping part of the invaded tracheal wall. However, the patient had neck postoperative subcutaneous emphysema within a week after the operation. The emphysema was clinically stable, and no further intervention was required. Subcutaneous emphysema disappeared after about one month. One patient had an axillary incision infection 4 days after the operation, and oral antibiotics were prescribed.

In the CH group, 8 (7.7%) temporary vocal cord paresis, 32 (30.8%) postoperative thyroid area traction, and 1 (0.96%) wound seroma were observed. All temporary vocal cord paresis were resolved in 3 months without any treatment. The patient with the wound seroma underwent conservative treatment and made a spontaneous recovery.

## Discussion

Robotic thyroidectomy is a new procedure that has shown early results that are at least similar to standard endoscopic techniques. Robotic thyroidectomy employing the gasless transaxillary approach, first published in 2007, may be considered a “scarless” alternative to standard open thyroidectomy for patients with thyroid cancer. Another obvious advantage of robotic surgery is the capacity to execute full thyroidectomy with extensive cervical lymph node dissection while maintaining dexterity and vision [8–10]. Learning new surgical skills is considered challenging, and the robot system is often seen as a complicated method with a lengthy learning curve. Many surgeons, however, have already indicate that the use of robots may enhance the learning curve of surgeons seeking to acquire sophisticated laparoscopic skills. Even for seasoned high-volume head and neck surgeons, the learning curve for traditional endoscopic unilateral lobectomy without lymph node dissection is projected to be about 60 cases [11]. According to recent research results, the learning curve for robot methods were shorter (range: 20–50 cases) than traditional endoscopic methods [12–14]. This indicates that the typical approach is constrained by assistant-dependent unstable camera platforms, two-dimensional views, restricted instrument maneuverability, and fixed instrument tips. Thus, we anticipate that the da Vinci robot system will enable surgeons to have a shorter surgical learning curve.

Prior research has indicated that the learning curve for robotic thyroidectomy may vary based on the technique used [15–18]. Owing to a comparable operational perspective, the midline approach is simpler than the usual open thyroidectomy [19]. By contrast, the lateral techniques—such as transaxillary or retroauricular approaches—may need more time and operation hours for the surgeon to be acquainted with the anatomy and procedure, particularly during contralateral side dissection in case of complete thyroidectomy [20]. Our study’s learning curve is similar to earlier research that used the midline method [21]. The current study found that the learning curve of operative time for gasless RATH extended to 31 cases for experienced, high-volume head and neck surgeons. CUSUM analysis and moving average graphs revealed a unique learning phase transition between 25 and 30 examples. In the comparisons between learning phase and succeeding phase, operational time and drainage volume were dramatically reduced. Our research additionally evaluated the gasless RATH patient group in terms of early cases (phase 1, 1–30), mid-level cases (phase 2, 31–60), and late cases (phase 3, 61–105), which correspond to before and beyond the learning curve’s endpoint. Once the learning curve phase was over, the operating timeframes and perioperative parameters of medium cases were comparable to those of the late cases, showing that the surgeon had learned the essential technical abilities to effectively execute robotic thyroid surgery by this time.

Blood loss and drainage volume have long been used as a proxy to assess the learning curve. These parameters likely indicate the capacity to sustain coagulation and the quality of tissue manipulation, where less blood loss may indicate operational proficiency [22]. In our research, although the CUSUM plot for drainage volume did not show a clear single peak, the drainage volume of phase 1 was greater than that of phase 2. There was no significant difference in blood loss among patients at different stages. This suggested that blood loss may potentially be impacted by patient-specific variables such as Hashimoto thyroiditis and hyperthyroidism, thereby diminishing its predictive ability for assessing the learning curve. However, the average total drainage during the first 48 postoperative hours in gasless RATH group (154.7 ± 42.9 mL) was more than that in the CH group (95.3 ± 30.1 mL). The possible reasons include larger surgical scope, longer operation time, and more exudate from the surgical passage in the former than the latter.

To objectively assess the impact of operation on parathyroid glands, postoperative calcium and PTH were analyzed. These individuals did not have postoperative permanent hypocalcemia or permanent low PTH, because they had hemithyroidectomy and two or more parathyroid glands retained. However, some patients still have postoperative hypocalcemia or low PTH, which may be because of the sensitivity of the parathyroid glands to transient ischemia. Differences in the incidence of low postoperative calcium (15.2%) or low postoperative PTH (14.4%) were not significant between the two groups. However, the mean postoperative calcium in the gasless RATH group (2.09 ± 0.08 mmol/L) was significantly lower than that in the CH group (2.11 ± 0.09 mmol/L, *P* = 0.018), and the mean postoperative PTH of the gasless RATH group (31.1 ± 9.5 pg/mL) was significantly more than that in the CH group (27.4 ± 10.0 pg/mL, *P* = 0.006). Overall, compared to CH, gasless RATH demonstrated non-inferiority of parathyroid protection; the likely reasons may be that the 3D field of view, and the multiarticulated and stable motions of robotic instruments made PTG preservation accurate. The procedure may help eliminate interference with the surgeon’s vision when using a robotic camera, even when additional devices are introduced in the same direction [23, 24]. The sense of postoperative thyroid area traction was lesser in the gasless RATH group (11 patients, 10.5%) than in the CH group (32 patients, 30.8%). This may be because we entered through the posterior space of the anterior cervical muscle, and the space between the strap muscles and skin was not opened in order to avoid anterior cervical cicatricial contracture. The subcutaneous nerve endings were completely preserved.

A different surgical outcome between the two groups was the number of retrieved lymph nodes, which was lower in the gasless RATH (3.3 ± 2.7) group than in the CH group (4.4 ± 3.4, *P* = 0.009). The higher proportion of patients with concurrent Hashimoto’s thyroiditis in the CH group (22.1%) compared to the RATH group (14.1%) may be the first contributing factor, leading to more reactive lymph nodes in the CH group. On the other hand, the majority of our enrolled patients were cN0 stage patients (93.8%) [25]. Studies have indicated that for cN0 early-stage well-differentiated thyroid cancer patients undergoing unilateral lobe resection, prophylactic central compartment lymph node dissection does not affect long-term prognosis. Furthermore, no residual suspicious metastatic lymph nodes were found in the central compartment during postoperative follow-up for all patients. Therefore, RATH does not adversely affect patient prognosis. However, the differences in lymph node metastasis in the two groups was not significant (30.5% vs. 31.7%, *P* = 0.845).

The robotic surgery system has the advantages of a 3D field of view magnified > 10 times, internal joints with 7 degrees of freedom, remote control, and filtering of the operator’s shaking, providing a precise operation platform for thyroid surgery [26, 27]. Moreover, the shorter learning curve and the ability to establish surgical channels under direct vision make the gasless RATH easier to master, especially for doctors with no endoscopy experience. However, due to the lack of tactile feedback from a robotic system, the surgeon adapts to this new surgical mode only after adequate clinical practice [28–30]. In summary, the gasless RATH approach is simple, safe, and can be easily mastered.

Our study has some limitations. The primary restriction is its retrospective nature, which introduces the possibility of prejudice. Second, the very small number of patients in the two groups may not adequately support our statistical findings. Third, we were unable to compare the two approaches in terms of surgeon experience, because robotic thyroidectomy is a more recent technique than conventional thyroidectomy and the surgeon in the study had no experience of endoscopic surgery. Fourth, sensation in the anterior chest region for patients in the RATH group was not assessed. Consequently, our findings must be interpreted with caution, and more trials in larger patient populations are necessary.

In conclusion, for surgeons without experience of endoscopic thyroidectomy, the learning curve length for gasless RATH using the gasless transaxillary technique was reported to be 31 cases, and it has a similar complication rate as conventional hemithyroidectomy. However, the use of robots in thyroid surgery is still in its early phase, and many concerns including the advantages of using technology remain unaddressed. More prospective randomized trials are needed to assess the real learning curve of a novice endoscopic surgeon for robotic thyroidectomy.

## Data Availability

The datasets used and/or analysed during the current study are available from the corresponding author on reasonable request.

## References

[1] Miccoli P, Berti P, Raffaelli M, Conte M, Materazzi G, Galleri D (2001). Minimally invasive video-assisted thyroidectomy. Am J Surg.

[2] Ciudad P, Manrique OJ, Bustos SS, Vargas MI, Reynaga C, Agko M, Huang TCT, Benites EF, Mayer HF, Forte AJ (2020). Combined microvascular breast and lymphatic reconstruction with deep inferior epigastric perforator flap and gastroepiploic vascularized lymph node transfer for postmastectomy lymphedema patients. Gland Surg.

[3] de Vries LH, Aykan D, Lodewijk L, Damen JAA, Borel Rinkes IHM, Vriens MR (2021). Outcomes of minimally invasive thyroid surgery - A systematic review and Meta-analysis. Front Endocrinol (Lausanne).

[4] Chang YW, Lee HY, Ji WB, Kim HY, Kim WY, Lee JB, Son GS (2020). Detailed comparison of robotic and endoscopic transaxillary thyroidectomy. Asian J Surg.

[5] Garstka ME, Alameer ES, Awwad SA, Kandil E (2019). Conventional robotic endoscopic thyroidectomy for thyroid Cancer. Endocrinol Metab Clin North Am.

[6] Kwak J, Kim SJ, Xu Z, Lee K, Ahn JH, Yu HW, Chai YJ, Choi JY, Lee KE. Robotic completion Thyroidectomy via the bilateral axillo-breast Approach. J Clin Med 2021, 10(8). 10.3390/jcm10081707 PMC8071380 33921046

[7] Tae K (2021). Robotic thyroid surgery. Auris Nasus Larynx.

[8] Sun GH, Peress L, Pynnonen MA (2014). Systematic review and meta-analysis of robotic vs conventional thyroidectomy approaches for thyroid disease. Otolaryngol Head Neck Surg.

[9] Pavlidis ET, Psarras KK, Symeonidis NG, Martzivanou EK, Nikolaidou CC, Stavrati KE, Pavlidis TE. Robot-Assisted Thyroidectomy Versus Open Thyroidectomy in the Treatment of Well Differentiated Thyroid Carcinoma. *JSLS* 2021, 25(3). 10.4293/JSLS.2021.00032 PMC8325479 34354333

[10] Shin IB, Bae DS. Comparison of the postoperative outcomes of the Mini-flap bilateral axillo-breast Approach (BABA) and Conventional BABA Robot-assisted thyroidectomy. J Clin Med 2022, 11(16). 10.3390/jcm11164894 PMC9410211 36013133

[11] Liu S, Qiu M, Jiang DZ, Zheng XM, Zhang W, Shen HL, Shan CX (2009). The learning curve for endoscopic thyroidectomy: a single surgeon’s experience. Surg Endosc.

[12] Ou YC, Yang CR, Wang J, Yang CK, Cheng CL, Patel VR, Tewari AK (2011). The learning curve for reducing complications of robotic-assisted laparoscopic radical prostatectomy by a single surgeon. BJU Int.

[13] Proietti F, La Regina D, Pini R, Di Giuseppe M, Cianfarani A, Mongelli F (2021). Learning curve of robotic-assisted transabdominal preperitoneal repair (rTAPP) for inguinal hernias. Surg Endosc.

[14] Patel VR, Tully AS, Holmes R, Lindsay J (2005). Robotic radical prostatectomy in the community setting–the learning curve and beyond: initial 200 cases. J Urol.

[15] Yu J, Rao S, Lin Z, Pan Z, Zheng X, Wang Z (2019). The learning curve of endoscopic thyroid surgery for papillary thyroid microcarcinoma: CUSUM analysis of a single surgeon’s experience. Surg Endosc.

[16] Lee DY, Oh DJ, Kang KR, Kim MS, Oh KH, Baek SK, Kwon SY, Woo JS, Jung KY (2016). Comparison of learning curves for Retroauricular and Transaxillary Endoscopic Hemithyroidectomy. Ann Surg Oncol.

[17] Luo JH, Xiang C, Wang P, Wang Y (2020). The learning curve for Transoral endoscopic thyroid surgery: a single surgeon’s 204 case experience. J Laparoendosc Adv Surg Tech A.

[18] Razavi CR, Vasiliou E, Tufano RP, Russell JO (2018). Learning curve for Transoral endoscopic thyroid lobectomy. Otolaryngol Head Neck Surg.

[19] Kim WW, Jung JH, Park HY (2015). The learning curve for robotic thyroidectomy using a bilateral axillo-breast Approach from the 100 cases. Surg Laparosc Endosc Percutan Tech.

[20] Bae DS, Koo do H, Choi JY, Kim E, Lee KE, Youn YK (2014). Current status of robotic thyroid surgery in South Korea: a web-based survey. World J Surg.

[21] Liu SY, Kim JS (2017). Bilateral axillo-breast approach robotic thyroidectomy: review of evidences. Gland Surg.

[22] Kassite I, Bejan-Angoulvant T, Lardy H, Binet A (2019). A systematic review of the learning curve in robotic surgery: range and heterogeneity. Surg Endosc.

[23] Kang SW, Lee SC, Lee SH, Lee KY, Jeong JJ, Lee YS, Nam KH, Chang HS, Chung WY, Park CS (2009). Robotic thyroid surgery using a gasless, transaxillary approach and the Da Vinci S system: the operative outcomes of 338 consecutive patients. Surgery.

[24] Stang MT, Yip L, Wharry L, Bartlett DL, McCoy KL, Carty SE (2018). Gasless Transaxillary Endoscopic Thyroidectomy with robotic assistance: a high-volume experience in North America. Thyroid.

[25] Kim SK, Woo JW, Lee JH, Park I, Choe JH, Kim JH, Kim JS (2016). Prophylactic Central Neck Dissection might not be necessary in papillary thyroid carcinoma: analysis of 11,569 cases from a single Institution. J Am Coll Surg.

[26] Matteucci V, Bai D, Fregoli L, Papini P, Aghababyan A, Docimo G, Miccoli P, Materazzi G (2021). The effect of robot-assisted transaxillary thyroidectomy (RATT) on body image is better than the conventional approach with cervicotomy: a preliminary report. Updates Surg.

[27] Lo EM, Kim HL (2021). Robot-assisted surgery for Upper Tract Urothelial Carcinoma. Urol Clin North Am.

[28] Ryu JM, Kim JY, Choi HJ, Ko B, Kim J, Cho J, Lee MH, Choi JE, Kim JH, Lee J (2022). Robot-assisted nipple-sparing Mastectomy with Immediate breast Reconstruction: an initial experience of the Korea Robot-Endoscopy Minimal Access breast surgery Study Group (KoREa-BSG). Ann Surg.

[29] Yuan W, Cao W, Meng X, Zhu H, Liu X, Cui C, Tao L, Zhu Y (2020). Learning curve of Robot-assisted Percutaneous Kyphoplasty for Osteoporotic Vertebral Compression fractures. World Neurosurg.

[30] Larcher A, Muttin F, Peyronnet B, De Naeyer G, Khene ZE, Dell’Oglio P, Ferreiro C, Schatteman P, Capitanio U, D’Hondt F (2019). The learning curve for Robot-assisted partial nephrectomy: impact of Surgical Experience on Perioperative outcomes. Eur Urol.

